# Vaccine strain *Listeria monocytogenes* abscess in a dog: a case report

**DOI:** 10.1186/s12917-019-2216-y

**Published:** 2019-12-21

**Authors:** Margaret L. Musser, Erika P. Berger, Cameron Parsons, Sophia Kathariou, Chad M. Johannes

**Affiliations:** 1 Iowa State University, Veterinary Clinical Sciences, 1809 South Riverside Drive, Ames, IA 50011 USA; 2 Department of Food, Bioprocessing and Nutrition Sciences, North Carolina State University, Raleigh, NC USA

**Keywords:** Canine, Osteosarcoma, Immunotherapy, *Listeria monocytogenes*

## Abstract

**Background:**

*Listeria monocytogenes* is a promising therapeutic vaccine vector for cancer immunotherapy. Although highly attenuated, three cases of systemic listeriosis have been reported in people following treatment with *Listeria*-based therapeutic vaccines. This complication has thus far not been reported in canine patients.

**Case presentation:**

A dog previously diagnosed with osteoblastic osteosarcoma was presented for care following administration of three doses of the Canine Osteosarcoma Vaccine-Live Listeria Vector. On routine staging chest radiographs, mild sternal lymphadenopathy and a right caudoventral thoracic mass effect were noted. Further evaluation of the mass effect with computed tomography and ultrasound revealed a cavitated mass associated with the 7th right rib. Aspirates of the mass cultured positive for *Listeria monocytogenes.* The mass and associated ribs were surgically removed. Histopathology was consistent with metastatic osteoblastic osteosarcoma. Treatment was continued with doxorubicin chemotherapy and at the time of publication, the dog was alive over 1 year following diagnosis with no evidence of further disease progression. Genotyping of the abscess-derived *L. monocytogenes* was consistent with the vaccine strain.

**Conclusions:**

This case represents the first veterinary case to describe development of a *Listeria* abscess following administration of a *Listeria*-based therapeutic vaccine.

## Background

Osteosarcoma is an aggressive, highly malignant, immunogenic cancer in both humans and dogs [1]. Both species lack effective, long-term treatment options, making novel immunotherapeutic strategies appealing [1]. An alternative immunotherapeutic approach with a HER2-targeting *Listeria* vaccine against canine OSA was reported in 2016 by Mason et al. [2] In this clinical study of 18 patients, the median survival time was significantly longer for those that received the vaccine in addition to standard-of-care amputation and chemotherapy (956 days) compared to a historical control group treated with standard-of-care alone (423 days; *P* = .014) [2]. In 2017, Aratana Therapeutics received a conditional license from the United States Department of Agriculture’s Center for Veterinary Biologics to conduct extended field studies to confirm the safety of the live *Listeria* vector product in hopes of being granted full product licensure.

*Listeria monocytogenes* is a facultative intracellular bacterium that is capable of activating strong CD8 and CD4 T-lymphocyte responses via dual presentation on major histocompatibility complex molecules class I and II [3]. When used in therapeutic vaccines, *Listeria* is alive but highly attenuated. In humans, three cases of systemic listeriosis have been reported where a live therapeutic vaccine was implicated [4–6]. To the authors’ knowledge, this potential complication has not been previously described in the veterinary literature.

## Case description

A 6-year-old, male-castrated English Pointer was presented to the Iowa State University Hixson-Lied Small Animal Hospital for evaluation following three planned administrations of the conditionally licensed Canine Osteosarcoma Vaccine-Live Listeria Vector (COV-LLV) (Aratana Therapeutics, Inc.). The dog had been diagnosed with osteoblastic osteosarcoma (OSA) of the right proximal humerus and had received right thoracic limb amputation and four doses of carboplatin (Hospira) chemotherapy (300 mg/m^2^ IV q3w). Four weeks following the last carboplatin chemotherapy treatment, the dog was enrolled in a clinical trial investigating the safety of the COV-LLV in dogs with appendicular OSA previously treated with standard-of-care amputation and chemotherapy. The patient received three planned doses of the vaccine, 3 weeks apart, with minimal toxicity. Prior to commencement of chemotherapy and prior to starting the COV-LLV, three-view chest radiographs were performed which were unremarkable at both time points.

The patient was presented 3 weeks following the final vaccine for evaluation. Upon presentation, the dog had a normal pulse (130 beats per minute), was panting with a normal respiratory effort, and was mildly febrile on rectal temperature (39.7 °C). He was bright, alert, and well-hydrated. Overall, the physical examination was unremarkable. Per the study protocol, a CBC and chemistry panel were obtained. The chemistry panel was unremarkable. The CBC revealed a mild monocytosis (2060/uL; reference range [RR] 150–1350/uL) and mild thrombocytopenia (119,000/uL; RR 200,000-500,000/uL). The dog was considered to have completed the study protocol and was officially taken off study.

Staging chest radiographs, as recommended by the attending oncologist but not as part of the study protocol, were completed. Although no pulmonary metastatic disease was noted, an approximately 8 cm long, right caudoventral mass effect and mild sternal lymphadenopathy were present. This was a significant change from the chest radiographs obtained 10 weeks previously which were radiographically normal. Given these unusual findings, ultrasound of the abdomen and chest were completed. The abdomen was found to be ultrasonographically normal. The chest ultrasound revealed a structure in the right caudoventral extrapleural space, with a thick, undulant hyperechoic wall containing a large amount of mildly echogenic fluid with mild peripheral vascularity on color Doppler. An uncomplicated aspirate of the right extrapleural mass was obtained. Several milliliters of dark, red, cloudy fluid were acquired and submitted for cytology and culture. Cytology of the fluid revealed several intracellular and extracellular bacteria, consistent with septic effusion and hemorrhage. Given the concern for developing sepsis, blood and urine cultures were obtained.

**Fig. 1:**
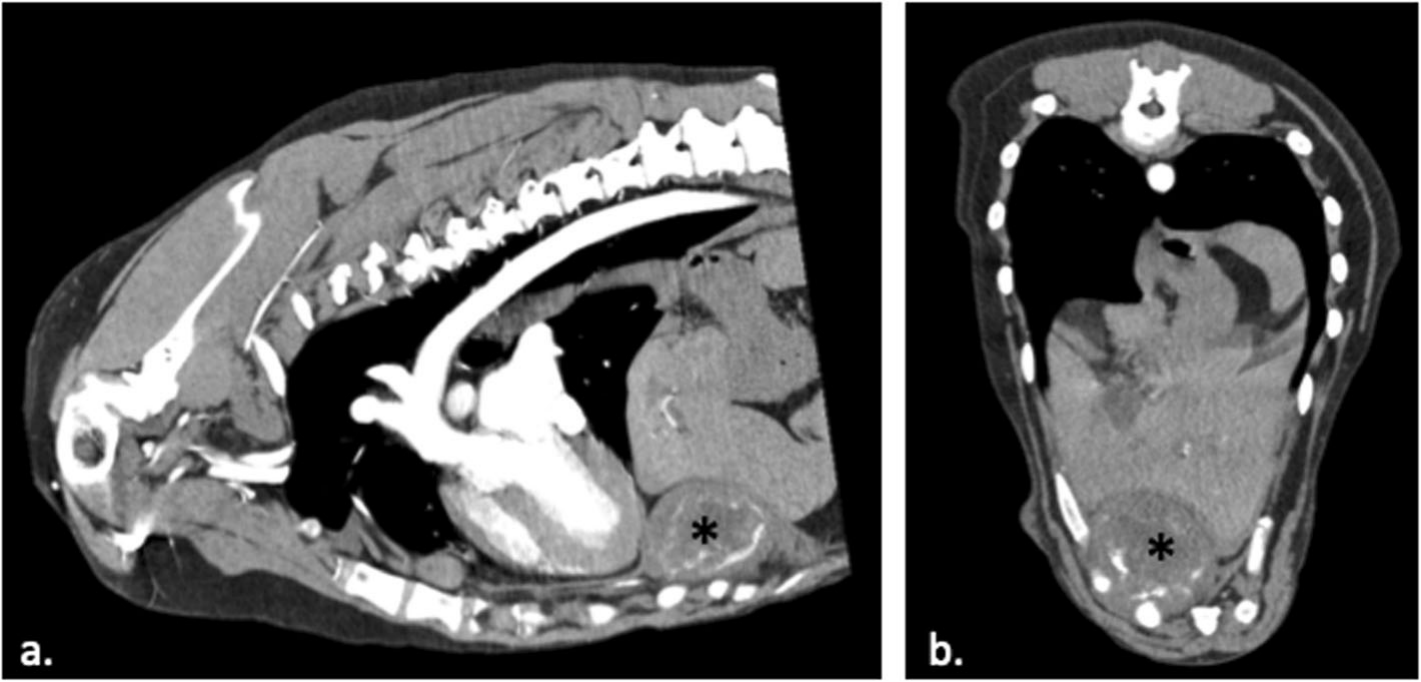
Thoracic computed tomography (CT) scan. **a.** Sagittal and **b.** transverse post-contrast thoracic CT scan in a patient with a vaccine-induced *Listeria monocytogenes* abscess (*)

The dog was hospitalized on supportive care including IV ampicillin/sulbactam (Pfizer; 30 mg/kg IV q 8 h) and oral cefpodoxime (Zoetis; 5.8 mg/k PO q 24 h) to provide broad-spectrum coverage, including against *Listeria monocytogenes*. An echocardiogram to evaluate for evidence of a vegetative lesion was completed and was normal. A computed tomography (CT) scan of the chest was completed for surgical planning to remove the suspected chest abscess. This revealed a heterogenous extrapleural mass dorsal to the xiphoid and adjacent to the right 7th costochondral junction (Fig. 1).

The dog was taken to surgery for removal of the abscess via a median sternotomy. The abscess, as well as the 6th and 7th costal portions of the right ribs were excised along with the internal muscle layer. The entire abscess and ribs were submitted for histopathology.

Culture from the original abscess fluid cytology was consistent with a *Listeria monocytogenes* abscess. Blood culture and urine culture were negative. Histopathology of the rib lesion was consistent with an incompletely excised osteoblastic OSA. Due to the metastatic lesion and lack of disease control, the dog was started on adjuvant doxorubicin (Pfizer; 30 mg/m2 IV q3w × 6). At last follow-up, the dog was alive and free of additional metastatic disease greater than 1 year after initial diagnosis.

The *Listeria* isolated from the abscess was sent to North Carolina State University for characterization. The strain cultured from the abscess was found to be *L. monocytogenes* of serotype 1/2a via multiplex serotype PCR, [7] which was also the serotype of the vaccine strain. The abscess strain was found to be streptomycin-resistant, which was also a property of the vaccine strain that is otherwise uncommon in *L. monocytogenes*. Lastly, PCR with primers Her-2-Chimera (F) and Her-2-Chimera (R) [8] confirmed the presence of a chimeric human Her2/neu genetic fusion in both the abscess strain and the vaccine strain [8]. As these genes are of human origin they are not expected to occur naturally in *L. monocytogenes* or in dogs, hence the presence of these nucleotide sequences in both strains supports the identity between the vaccine and the abscess strain.

## Discussion and conclusions

The dog described herein developed a confirmed vaccine strain *Listeria* abscess following treatment with the COV-LLV. It is of note that the abscess in this case developed at an area of metastatic disease. Although this localization has not been reported previously, in one human case report intravenously-infused *Listeria*-based vaccine was hypothesized to have infected tissues and/or prosthetic material at recent fracture sites following unrelated trauma [6]. It was hypothesized that since fracture repair undertaken by the body suppresses the local immune response, these sites functioned as a protected niche for *Listeria* [6]. A fracture was not reported to be associated with the metastatic rib lesion reported in this case. However, decreased immune surveillance at the metastatic site, which has been shown to be present in human OSA, [9] may have provided a niche for the *Listeria* to grow uninhibited.

One concern raised by this case is the fact that a highly-attenuated bacterium caused disease. The *Listeria* vaccine given to the current patient has a reduction in virulence compared to previous iterations of *Listeria-*based vaccines due to deletion of a major *Listeria* virulence gene (*actA*) that is necessary for the cell-to-cell spread of the bacterium [10]. Although this attenuation of *Listeria* disrupts the intracellular lifecycle, it leaves other virulence characteristics intact, including the ability of the *Listeria* to enter antigen-presenting cells via phagocytosis and replicate intracellularly, [2] and thus *Listeria* may still be able to move and replicate within a host.

Although the canine patient in this case had received chemotherapy previously, he was not considered to be immunocompromised at the time of vaccine administration. Immunization with live vaccines is not advised for immunocompromised people, [11] but timing and guidelines for appropriate use of live therapeutic vaccines in cancer patients have not been established and warrant significant attention due to the potential severe adverse effects of developing a vaccine-induced infection.

The abscess in this case was detected 3 weeks after the final vaccine administration. In the reported human cases, infection was present within hours, [5] days, [4] and 31 months [6] following vaccination. These limited data suggest that adverse repercussions from *Listeria* therapeutic vaccination could occur at any time following vaccination, including many months later, especially if there is an inviting biological niche to harbor the bacteria. This implies that prolonged, vigilant monitoring following therapeutic vaccination is required.

Early intervention in this case with appropriate antibiotics active against *Listeria* and removal of the abscess resulted in a positive outcome for this dog. However, this case highlights the required caution when using live, attenuated therapeutic vaccinations, especially in potentially immunocompromised individuals. It is important to stay attentive throughout treatment and remain alert to the potential for listeriosis-associated sepsis or other outcomes following treatment to direct timely and appropriate therapy. It also implies the importance of attention to handling of these products in veterinary medicine, as the vaccine and the treated patient may confer a zoonotic concern for veterinary professionals and family members that share their home with the patient (evidence of bacterial shedding by vaccine-treated patients has not been documented or fully investigated). This would be especially true for those who may themselves be immunocompromised.

## Data Availability

The datasets used and/or analysed during the current study are available from the corresponding author on reasonable request.

## Abbreviations

COV-LLV: Canine Osteosarcoma Vaccine-Live Listeria Vector
CT: Computed Tomography
OSA: Osteosarcoma
RR: Reference Range
